# Aflibercept 8 mg in neovascular age-related macular degeneration and diabetic macular oedema: Translating clinical trials into clinical practice

**DOI:** 10.1038/s41433-026-04779-5

**Published:** 2026-08-07

**Authors:** Sobha Sivaprasad, Michael W. Stewart, Charles C. Wykoff, David Eichenbaum, Jean-Francois Korobelnik, Paolo Lanzetta, Varun Chaudhary, Chui Ming Gemmy Cheung, Richard Gale, Marion R. Munk, Taiji Sakamoto, Andreas Stahl, Javier Zarranz-Ventura, Tien Yin Wong, Frank G. Holz, Anat Loewenstein

**Affiliations:** 1 NIHR Biomedical Research Centre, Moorfields Eye Hospital NHS Foundation Trust, London, UK; 2 Department of Ophthalmology, Mayo Clinic, Jacksonville, FL USA; 3 Retina Consultants of Texas, Retina Consultants of America, Houston, TX USA; 4 Retina Vitreous Associates of Florida, Saint Petersburg, FL USA; 5 Morsani College of Medicine at the University of South Florida, Tampa, FL USA; 6 CHU Bordeaux, Service d’Ophtalmologie, Bordeaux, France; 7 University of Bordeaux, INSERM, BPH, UMR1219 Bordeaux, France; 8 Department of Medicine-Ophthalmology, University of Udine, Udine, Italy; 9 Istituto Europeo di Microchirurgia Oculare-IEMO, Udine, Italy; 10 Department of Health Research Methods, Evidence and Impact, McMaster University, Hamilton, ON Canada; 11 Division of Ophthalmology, Department of Surgery, McMaster University, Hamilton, ON Canada; 12 Singapore Eye Research Institute, Singapore National Eye Centre, Singapore, Singapore; 13 Duke-National University of Singapore Medical School Singapore, Singapore, Singapore; 14 Hull York Medical School, University of York, York, UK; 15 Hull York Medical School, University of York, and York and Scarborough Teaching Hospitals NHS Foundation Trust, York, UK; 16 Gutblick Practise Group, Pfäffikon, Switzerland; 17 Department of Ophthalmology, Inselspital, Bern University Hospital, University of Bern, Bern, Switzerland; 18 Department of Ophthalmology, Northwestern University, Feinberg School of Medicine, Chicago, IL USA; 19 Department of Ophthalmology, Kagoshima University, Kagoshima, Japan; 20 Department of Ophthalmology, University Medicine Greifswald, Mecklenburg-Vorpommern, Greifswald, Germany; 21 Hospital Clínic de Barcelona, Barcelona, Spain; 22 Department of Surgery and Medical-Surgical Specialties, Faculty of Medicine, and Health Sciences, Universitat de Barcelona, Barcelona, Spain; 23 Institut d’Investigacions Biomèdiques August Pi i Sunyer (IDIBAPS), Barcelona, Spain; 24 Beijing Visual Science and Translational Eye Research Institute (BERI), Beijing Tsinghua Changgung Hospital, Beijing, School of Clinical Medicine, Tsinghua Medicine, Tsinghua University, Beijing, China; 25 School of Biomedical Engineering, Tsinghua Medicine, Tsinghua University, Beijing, China; 26 Department of Ophthalmology and GRADE Reading Center, University of Bonn, Bonn, Germany; 27 Department of Ophthalmology, Tel Aviv Medical Centre, Tel Aviv, Israel

**Keywords:** Therapeutics, Drug therapy

## Abstract

Provide expert-informed guidance on the integration of aflibercept 8 mg into routine clinical care for patients with neovascular age-related macular degeneration (nAMD) and diabetic macular oedema (DMO), based on clinical trial and real-world evidence. Sixteen retina specialists (‘experts’) met during the Association for Research in Vision and Ophthalmology (ARVO) annual meeting in May 2025 to discuss the clinical use of aflibercept 8 mg. A post-meeting online platform was used to collect additional independent input. Agreement was captured using binary responses and categorised as: all agreed (100%), majority agreed ( ≥ 75%), most agreed (50–74%), or non-consensus ( ≤ 50%). All experts agreed that aflibercept 8 mg may be considered as a first-line treatment in treatment-naïve patients with nAMD and DMO, and switching to aflibercept 8 mg can be considered for previously treated patients. The majority of experts agreed that switching patients with well-controlled disease on shorter or intermediate dosing intervals may enable further interval extension while maintaining disease control, and switching patients with poorly controlled disease may optimise anatomical outcomes and improve stability. Most experts agreed that three initial monthly loading doses are appropriate in newly diagnosed patients. No consensus was reached on optimal dosing-decision strategies after switching in patients with poorly controlled disease, nor on the timing of initial interval modification during treatment initiation. These expert opinions, informed by available, emerging clinical evidence and real-world clinical experience, support aflibercept 8 mg as a first-line and switch option for nAMD and DMO, using personalised treatment approaches for both treatment-naïve and previously treated patients.

## Introduction

The rapidly ageing global population, together with the epidemic of diabetes mellitus resulting from urbanisation and lifestyle-induced changes [1, 2], underscores the importance of managing the increasing number of patients with neovascular age-related macular degeneration (nAMD) and diabetic macular oedema (DMO). Achieving and maintaining good vision in affected patients is an important determinant of overall quality of life and well-being, social inclusion and economic productivity [3].

Therapies that inhibit the action of vascular endothelial growth factor (VEGF) substantially reduce the incidence of severe vision loss and blindness [4, 5], and have become the established standard of care for both nAMD and DMO [6–12]. The need for frequent intraocular injections, however, places a considerable burden on patients, their caregivers, physicians and other clinical staff, and the overall delivery of national health services [13, 14]. Long-term management of nAMD and DMO is often complicated by patient non-compliance, which challenges the routine delivery of effective, sustained care [15–17]. As a result, recent pharmacologic advances have focused on reducing anti-VEGF treatment burden while maintaining long-term vision outcomes.

Aflibercept 2 mg has been a long-standing established treatment for both nAMD and DMO and used globally since the 2010s. In the past few years, a higher-concentration formulation of aflibercept (aflibercept 8 mg) has been developed to deliver a four-fold higher molar dose (compared with aflibercept 2 mg) into the vitreous [18, 19]. Aflibercept 8 mg has been approved in various regions of the world, including North America, Europe, and Asia, for the treatment of patients with nAMD and DMO, based on positive data from the PULSAR phase 3 trial in patients with nAMD and the PHOTON phase 2/3 trial in patients with DMO [20, 21]. During the first year, aflibercept 8 mg is administered via extended treatment intervals of up to every 16 weeks, following three initial monthly doses [22–24]. The 2-year data from the PULSAR and PHOTON trials demonstrated that further extended treatment intervals may be considered in patients with stable visual and anatomical outcomes; accordingly, the FDA label was expanded to allow dosing intervals of up to 20 weeks, and the labels in the EU and other regions were expanded to allow dosing intervals of up to 24 weeks [22, 23].

In the pivotal clinical trials, in both the PULSAR (nAMD) and PHOTON (DMO) trials, aflibercept 8 mg achieved sustained visual and anatomical improvements through 156 weeks. In PULSAR, 40% of patients achieved ≥20-week intervals and 24% of patients achieved 24-week intervals by Week 156. Among patients who switched from aflibercept 2 mg to 8 mg at Week 96, 43% and 16% were treated on ≥16- and ≥20-week intervals by study end, respectively. In PHOTON, 48% and 28% of patients receiving aflibercept 8 mg from baseline achieved ≥20-week and 24-week intervals, respectively, by Week 156. Patients who switched from aflibercept 2 mg to 8 mg after Week 96 experienced substantially slower fluid re-accumulation compared with their previous rate with aflibercept 2 mg. In many cases, patients who switched doses were able to maintain disease stability while also extending their dosing intervals. The criteria for interval extension were consistent with clinical practice requiring stable visual acuity with no evidence of persistent or recurrent disease on optical coherence tomography (OCT). In both trials, the long-term safety profile was similar to that previously established for aflibercept 2 mg [20, 21, 25, 26].

Emerging real-world evidence complements these pivotal-trial findings. The ongoing global SPECTRUM phase 4 study, the largest real-world investigation of aflibercept 8 mg to date, is evaluating its effectiveness and safety across diverse populations of treatment-naïve and pre-treated patients with nAMD and DMO [27–31]. Thus far, published SPECTRUM data have shown early visual acuity gains in treatment-naïve patients with nAMD, together with improvements in central retinal thickness and retinal fluid outcomes in both treatment-naïve and previously treated cohorts by Week 8 [31]. Further results are expected to provide valuable insights into the effectiveness, safety, durability and utility of aflibercept 8 mg in routine clinical practice. In addition to the SPECTRUM trial, numerous investigator-initiated real-world studies evaluating the use of aflibercept 8 mg in routine clinical practice are currently underway [32, 33].

While regulatory approvals are based on pivotal trials, clinicians increasingly require practical guidance on patient selection, switching strategies and interval management that extends beyond trial protocols and real-world experience.

This article aims to provide a position document on the use and integration of aflibercept 8 mg into routine care for patients with nAMD and DMO. The recommendations presented are based on current clinical evidence with feedback and insight from 16 internationally recognised retina specialists from 12 countries.

## Methods

A two-phase, iterative expert consultation process was employed with the aim of collating expert guidance regarding the integration of aflibercept 8 mg into routine care for patients with nAMD and DMO. This process was not intended to generate formal consensus recommendations or clinical guidelines. Rather, it aimed to capture areas of alignment and divergence in expert clinical practice to inform clinicians navigating early adoption of aflibercept 8 mg.

These statements were presented to a group of global retina specialists (referred to hereafter as ‘experts’) at a roundtable meeting held in May 2025 alongside the annual meeting of the Association for Research in Vision and Ophthalmology (ARVO). The group comprised 16 experts from 12 countries: Canada, China, France, Germany, Israel, Italy, Japan, Singapore, Spain, Switzerland, the United Kingdom, and the United States.

Following discussion at the roundtable meeting, the statements were formalised for agreement and uploaded to Within3 (Lakewook, OH, USA), a collaborative online platform that enables feedback to be obtained asynchronously and analysed both quantitatively and qualitatively [34]. Experts were presented with 22 discrete questions within the platform and blinded to others’ responses in order to minimise potential influence and bias. Binary response options were programmed for questions requesting agreement with a particular statement or clinical scenario (i.e. yes/no or agree/disagree) to facilitate quantification of agreement as all agreed (100% agreement), majority agreed ( ≥ 75% agreement), most agreed (51–74% agreement), or non-consensus ( ≤ 50% agreement). Corresponding agreement thresholds for the 16-member panel were 16/16, 12–15/16, 9–11/16, and ≤ 8/16, respectively.

Follow-up questions allowing open text responses were also included to enable provision of further expert guidance and context. All questions were programmed as mandatory and could not be skipped.

## Results

Expert responses have been included in Table 1; the specific questions posed to experts are included in the Supplementary Information (Supplementary Table 1).

**Table 1:**
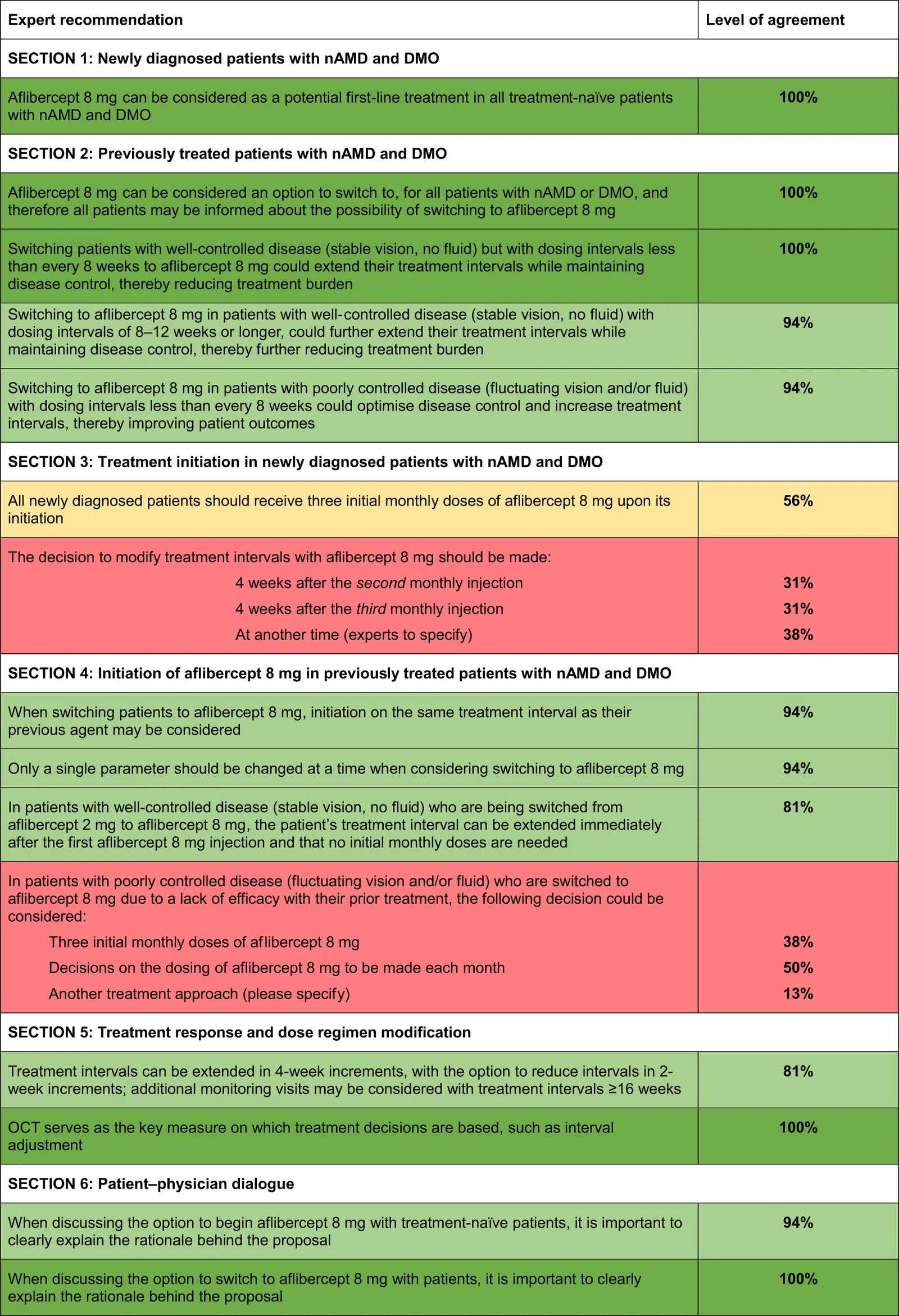
Expert recommendations.

Agreement captured using binary responses were categorised as: all agreed (100%,), majority agreed ( ≥ 75%,), most agreed (51–74%,), or non-consensus ( ≤ 50%,).

### SECTION 1: Newly diagnosed patients with nAMD and DMO

***Statement 1:*** **All experts agreed that aflibercept 8 mg can be considered as a first-line treatment in treatment-naïve patients with nAMD and DMO**

Experts described aflibercept 8 mg as an effective and durable therapy well suited for first-line use because it rapidly achieves clinical improvements that can be sustained to optimise long-term patient outcomes. Starting treatment with aflibercept 8 mg was viewed as a proactive strategy to minimise initial undertreatment, reduce early switching, and enhance adherence. Several experts highlighted that aflibercept 8 mg often enables early extension of the dosing intervals, thereby making it especially valuable for patients who may struggle with frequent clinic visits. By combining strong early efficacy with reduced treatment burden, aflibercept 8 mg was seen to align with the evolving need for personalised, sustainable care in nAMD and DMO.

**There are a few instances in which some experts may exercise clinical caution before considering first-line aflibercept 8 mg in newly diagnosed patients**

While 44% of experts reported no clinical reasons for caution when initiating first-line aflibercept 8 mg in newly diagnosed patients, others identified specific situations warranting caution in this patient group.

The most cited scenario was advanced or uncontrolled glaucoma, particularly in patients with large cup-to-disc ratios (C/D > 0.8), where transient intraocular pressure (IOP) elevations from any intravitreal injection may pose risk of further disc damage and progression of glaucoma.

Other situations in which some experts would exercise caution were noted. For patients with large pigment epithelial detachments, defined here as maximum height > 400 μm, the higher dose of aflibercept 8 mg compared with 2 mg could theoretically pose a risk of retinal pigment epithelium tears – although this is not evident from the pivotal trial data. Other class-related scenarios included the treatment of patients with a history of intraocular inflammation or hypersensitivity following aflibercept 2 mg in either eye; or simultaneous bilateral treatment in patients with DMO who have experienced recent cardiovascular events, where theoretical risk due to intravascular VEGF suppression might be a concern. Bilateral treatment may increase systemic drug exposure; and because there is limited safety data in patients with a recent ( ≤ 6 months) history of cardiovascular events, caution may be exercised in these patients [23].

### SECTION 2: Previously treated patients with nAMD and DMO

***Statement 2:*** **All experts agreed that switching to aflibercept 8 mg can be considered for all patients with nAMD or DMO, and, therefore, all patients may be informed about the possibility of switching to aflibercept 8 mg**

The panel highlighted that the decision of when to switch to aflibercept 8 mg is driven by specific clinical objectives and individual patient factors. For improved durability, experts reported switching to aflibercept 8 mg when patients on first-generation anti-VEGF agents were unable to extend treatment intervals beyond 12–16 weeks. For improved efficacy, some experts considered a switch to be appropriate if fluid persisted after the loading phase with another agent, given the importance of early disease control as a predictor of long-term outcomes [35]. Finally, to improve stability, a subset of clinicians described switching to aflibercept 8 mg after multiple failed attempts to extend treatment intervals with another agent, particularly when fluctuating fluid levels are observed.

***Statement 3:*** **All experts agreed that switching patients with well-controlled disease (stable vision, no fluid) but with dosing intervals less than every 8 weeks to aflibercept 8 mg could extend their treatment intervals while maintaining disease control, thereby reducing treatment burden**

Over two-thirds (69%) of experts highlighted the durability of aflibercept 8 mg to achieve a more sustainable treatment plan with lower treatment burden for patients. The experts noted that patients who respond to treatment and have well controlled disease with aflibercept 2 mg (or another anti-VEGF agent) every several weeks may be able to switch to aflibercept 8 mg and maintain good disease control. Clinical experience, however, has shown considerable inter-patient variability in achieving treatment intervals; while the extent and durability of treatment interval extension are primarily driven by underlying disease activity, the specific extension strategy, including the size of incremental extensions and the maximum interval attempted, may vary according to physician practice and patient-specific considerations.

***Statement 4:*** **The majority of experts agreed that switching to aflibercept 8 mg in patients with well-controlled disease (stable vision, no fluid) with dosing intervals of 8–12 weeks or longer, could** ***further*** **extend their treatment intervals while maintaining disease control, thereby** ***further*** **reducing treatment burden**

It was acknowledged that patient and clinician willingness to switch may be lower in this group, since both may feel comfortable with current dosing intervals. Shared decision-making, however, was agreed to be an essential component of patient care. For patients and caregivers who still experience an onerous burden even with less frequent injections, switching to aflibercept 8 mg could provide additional relief by further extending dosing intervals, while maintaining disease control.

This group of patients may present an ideal opportunity for exploring longer intervals (20–24 weeks), where clinically appropriate. As the clinical experience with aflibercept 8 mg continues to grow, clinicians are likely to become more confident switching patients, even those already stable on 16-week intervals. One expert noted that although there is currently limited evidence and experience with these patients, switching could be discussed if current dosing intervals are a substantial burden.

***Statement 5:*** **The majority of experts agreed that switching to aflibercept 8 mg in patients with poorly controlled disease (fluctuating vision and/or fluid) with dosing intervals less than every 8 weeks could optimise disease control and increase treatment intervals, thereby improving patient outcomes**

In general, the experts considered the higher molar dose of aflibercept 8 mg to be a likely contributor to improved anatomical control in some patients. However, responses in poorly controlled or refractory eyes can be heterogeneous, and the ability to extend treatment intervals should be considered only once stability is achieved, guided by clinical judgement and appropriate monitoring.

Even if some patients do not reach 8-week intervals, many may still experience improved anatomical stability after switching to aflibercept 8 mg. Nevertheless, outcomes may vary, and treatment goals should prioritise achieving and maintaining disease control, given that poor or fluctuating disease control has been consistently linked to poorer visual outcomes.

### Some experts noted a few additional patient profiles who could benefit from a switch to aflibercept 8 mg

Experts anticipate a shift from the current standard of care to aflibercept 8 mg for improved durability and outcomes in patients with polypoidal choroidal vasculopathy. Opinions are informed by the evolving evidence, including findings from the subgroup of patients with polypoidal choroidal vasculopathy in the PULSAR trial [36]. In addition, among patients with DMO with a severe disease status, a switch to aflibercept 8 mg may lead to timely improvement, including slower fluid reaccumulation [37, 38]. Lastly, patients for whom frequent clinic visits are not feasible due to comorbidities, geographical distance, or caregiver dependence may benefit from treatment with aflibercept 8 mg, since this could reduce treatment burden.

### SECTION 3: Treatment initiation in newly diagnosed patients with nAMD and DMO

***Statement 6:*** **Most experts recommend that all newly diagnosed patients may receive three initial monthly doses of aflibercept 8 mg, in agreement with the product label and PULSAR and PHOTON study design**

While over half of experts agreed with this recommendation, a substantial proportion (44%) shared that in routine practice, they adopt a more flexible, response-guided approach, particularly where patients are closely monitored. If the retina is dry and visual acuity is stable after one or two injections, some clinicians may initiate treat-and-extend protocols sooner to avoid further loading and overdosing in select patients, although this approach may not be consistent with the product label in all countries. Ongoing studies may help to clarify the minimum loading requirements for aflibercept 8 mg in DMO and nAMD, thereby augmenting the personalisation of future treatment strategies.

***Statement 7:*** **Agreement was not reached on when to first consider modifying the treatment interval after initiating aflibercept 8 mg**

Panel responses revealed an approximate three-way split in timing preferences. Some experts explained that they prefer to assess disease activity and adjust intervals 4 weeks after the third loading injection, aligning closely with the PULSAR and PHOTON trial protocols. This timing allows for a full evaluation of the three-dose loading phase and supports practical scheduling for both the patient and clinic. Alternatively, some experts begin assessing patients at the time of, or shortly after, the second or third injection, especially if the patient shows an excellent response. In such cases, early extension to 8-weekly intervals and beyond may then be pursued to minimise treatment burden while maintaining disease control. Finally, some experts prefer alternative timings, such as 6–8 weeks after the third injection or once maximal anatomical and visual improvement is observed. These experts emphasise the importance of allowing sufficient time to evaluate durability, particularly in nAMD versus DMO.

The panel generally felt that the *per label* guidance to consider the ongoing injection regimen strategy at the time of the third injection in patients treated with three loading doses of aflibercept 8 mg was reasonable [22, 23].

### SECTION 4: Initiation of aflibercept 8 mg in previously treated patients with nAMD and DMO

***Statement 8:*** **The majority of experts agreed that, when switching patients to aflibercept 8 mg, beginning with the same treatment interval as their previous agent may be considered**

When switching patients to aflibercept 8 mg, starting with the same interval as the previous treatment is generally appropriate, particularly in patients who are stable on their current schedule. This approach maintains patient trust and avoids what may be considered unnecessary treatment burden, given that the patient has probably already undergone a loading phase with another agent.

Maintaining the same dosing interval may also provide a baseline to assess patient response to aflibercept 8 mg. If disease control improves or remains stable, clinicians may then consider extending the interval with aflibercept 8 mg, thereby leveraging its longer durability. In some cases, particularly where the prior treatment failed to sufficiently control disease activity, clinicians may choose to either maintain or modestly reduce the interval, depending on the patient’s response and the clinician’s judgement.

There may be exceptions to maintaining the same dosing interval when switching to aflibercept 8 mg. For example, if a patient is already on a dosing interval of 8–12 weeks due to limited treatment responsiveness, and the clinician has determined that a more intensive approach with the current therapy will not be suitable, the patient may benefit from a short-term interval reduction upon switching to aflibercept 8 mg, to assess efficacy. Conversely, if a patient is well-controlled and switched primarily for durability, some clinicians may choose to extend the interval as part of, or directly after, switching.

***Statement 9:*** **The majority of experts agreed that only a single parameter should be changed at a time when considering switching to aflibercept 8 mg**. ***For example, if the agent is switched, the treatment interval should be kept the same***

When switching to aflibercept 8 mg, it is generally advisable to maintain the existing treatment interval for at least one dosing interval. This allows the clinician to isolate and determine the efficacy of aflibercept 8 mg without introducing confounding variables. Reasonable exceptions to this principle might include patients who have good disease control on their current treatment, in whom it may be clinically appropriate to both switch to aflibercept 8 mg and trial a modest extension simultaneously. This is the case when the switch is driven not by inadequate response, but by a desire to reduce treatment burden. In contrast, for patients with an inadequate treatment response or unstable disease, the treatment interval can be maintained initially to ensure the efficacy of aflibercept 8 mg before attempting any extension.

***Statement 10:*** **The majority of experts agreed that patients with well-controlled disease (stable vision, no fluid) who are being switched from aflibercept 2 mg to aflibercept 8 mg may not require initial monthly doses and may reasonably consider early interval extension in selected stable patients, guided by shared decision-making and close monitoring**

Given that aflibercept 2 mg and 8 mg share the same mechanism of action, switching may not require re-loading when disease control has already been established. In this context, some experts may reasonably consider early interval extension in selected stable patients, with close monitoring and shared decision-making. Others may prefer to maintain the pre-switch interval for at least one dosing cycle before attempting extension, particularly in patients previously managed on very short intervals or where there is residual concern about durability.

These approaches reflect variation in real-world clinical practice, recognising that prospective evidence specifically evaluating early interval extension immediately after switching remains limited.

***Statement 11:*** **Agreement was not reached on dosing decisions in patients with poorly controlled disease (fluctuating vision and/or fluid) who are switched to aflibercept 8 mg due to a lack of efficacy with their prior treatment**

Month-to-month decision-making (where clinic capacity permits) allows agile treatment adjustments in these patients who often require repeated treatment to achieve visual and anatomical improvement. However, approximately a third of experts would prefer to decide after three initial monthly doses, viewing this as a pragmatic strategy in patients with dosing intervals of <8 weeks.

In addition, several experts advocated for an approach tailored to the patient. For example, some would initially match the prior injection interval, then assess after the first aflibercept 8 mg dose whether extension or shortening is warranted. It was also noted that in patients with longstanding suboptimal response across multiple agents, the diagnosis itself may be re-evaluated with additional imaging.

### SECTION 5: Treatment response and dose regimen modification

***Statement 12:*** **Most experts agreed that treatment intervals can be extended in 4-week increments, with the option to reduce intervals in 2-week increments. Additional monitoring visits may be considered with treatment intervals** ≥ **16 weeks**

Extending dosing intervals in 4-week increments was considered a reasonable approach in treatment-naïve patients receiving aflibercept 8 mg. Though some experts noted that they would more likely offer 2-week extensions in certain scenarios, including when a patient is being switched to aflibercept 8 mg; if they present with severe disease that is known to require frequent dosing; or if extending further than 8-week dosing intervals. It was further noted that the durability profile of aflibercept 8 mg observed in the PULSAR and PHOTON trials, in which many patients achieved and maintained ≥ 12-week intervals following the initial monthly dosing period, provides greater confidence to extend intervals more decisively where disease control is stable.

There was some divergence in opinion regarding the need for additional monitoring visits at longer intervals (≥ 16 weeks), with some experts expressing that a pure treat-and-extend approach would not be expected to require additional monitoring between injections. Alternatively, some experts prefer monitoring approximately every 12–16 weeks, which includes observing the fellow eye.

### Some experts acknowledged that immediate extension to 16-week intervals may be limited to select patient cases

Immediate extension to 16-week intervals (after three loading doses) may be appropriate for patients achieving rapid disease control during the loading phase or after the first dose of aflibercept 8 mg, but some experts prefer to assess the patient’s response to treatment before extending. Most experts prefer to step through at least a 12-week treatment interval before extending to a 16-week interval, to maintain the peak response while reducing the risk of disease recurrence. Prospective studies are still needed to define optimal monitoring strategies at intervals beyond 16 weeks.

For patients who began treatment with mild DMO and achieved rapid fluid resolution and normal visual acuity, some of the panel reported greater confidence in directly extending to 16-week intervals. In younger, working-age patients, the risk of visual loss is low, and minimising clinic visits is an important consideration. Physicians may wish to consider diabetic retinopathy severity in their strategy for treatment and monitoring intervals.

***Statement 13:*** **All experts agree that OCT serves as the key measure on which treatment decisions, such as interval adjustment, are based**

Published literature supports the conviction that OCT provides objective, reproducible and robust measurements over time in both nAMD and DMO. Though OCT measurements are indispensable for managing these patients, treatment decisions for DMO are also based on examination, photography and/or angiography.

### While clinical experience supports fewer clinic visits for well-controlled patients on extended intervals, monitoring strategies can remain tailored to patient needs

Patients who have been gradually extended to intervals of 20–24 weeks likely have well-controlled disease, so disease monitoring only at the time of injections may be sufficient. However, a subset of experts advocated for an interim evaluation, typically halfway between injections (i.e. a monitoring visit at Week 10–12 for patients on intervals of 20–24 weeks), particularly in patients with monocular vision, those recently extended to these intervals and who have not yet demonstrated long-term disease control, or those without access to self-monitoring tools (e.g. home OCT or Amsler grid testing). Other experts emphasised patient education and shared responsibility instead of additional monitoring visits. The status of the fellow eye should be considered, since this may warrant shorter monitoring intervals.

### SECTION 6: Patient–physician dialogue

***Statement 14:*** **Most experts agreed that when discussing the option to begin aflibercept 8 mg with treatment-naïve patients, it is important to clearly explain the rationale behind the proposal**

When discussing treatment goals with patients, some experts noted the value of emphasising the importance of early fluid control to preserve visual function. Visual acuity and OCT imaging can be used to help patients understand their disease state and the importance of early disease control. Clinicians may also draw on their extensive experience with aflibercept 2 mg when introducing aflibercept 8 mg as an option with the potential for earlier extension of treatment intervals. Selected data from the PULSAR and PHOTON trials, alongside emerging real-world evidence, can be used to illustrate the clinical benefits and safety of aflibercept 8 mg relative to aflibercept 2 mg, as well as the higher proportion of patients achieving extended dosing intervals at 2 years [20, 21].

***Statement 15:*** **All experts agreed that when discussing the option to switch to aflibercept 8 mg with patients, it is important to clearly explain the rationale behind the proposal**

Framing aflibercept 8 mg as a higher-dose version of aflibercept 2 mg can be reassuring, since this is a drug that patients already know, and the available evidence on aflibercept 8 mg supports a comparable safety profile to that of aflibercept 2 mg. This can be shown with graphics showing drug durability curves. Reviewing OCT scans with patients that show both improvements and ongoing needs can also be helpful. Printed materials and informational leaflets were seen as valuable, particularly those that highlight longer intervals with aflibercept 8 mg (e.g. the aflibercept 2 mg and 8 mg leaflets). Furthermore, experts suggested that a short, patient-friendly video playing in waiting rooms would help explain important clinical information.

## Discussion

This article provides insight and guidance on the integration of aflibercept 8 mg as both a first-line therapy and a ‘switch’ option for the treatment of nAMD and DMO. High levels of agreement across multiple recommendations underscore the collective confidence in the efficacy, durability and safety profile of aflibercept 8 mg in both treatment-naïve and previously treated populations, and is supportive of other regional guidance [20–24, 39]. Although this manuscript reflects the views of an international group of retina specialists, reimbursement policies, regulatory labelling and treatment paradigms for aflibercept 8 mg may vary across regions. Accordingly, the recommendations presented should be interpreted within the context of local clinical practice and healthcare system constraints.

Among treatment-naïve patients, the unanimous endorsement of aflibercept 8 mg as a first-line treatment option reflects the strong efficacy and acceptable safety profile demonstrated in the PULSAR and PHOTON trials [20, 21, 25, 26]. These trials enroled diverse patient populations and showed that aflibercept 8 mg rapidly resolved fluid with long-term visual and anatomical benefits. The importance of initiating treatment with a potent and durable agent is reinforced by findings from DRCR.net protocols I, T and V, which demonstrated that persistent or fluctuating fluid during the first year of anti-VEGF therapy correlates with poorer long-term vision outcomes [40–42]. Moreover, multiple studies show that early visual acuity responses predict better long-term results [43–45], thereby supporting the clinical value of aflibercept 8 mg as a first-line therapy in DMO and nAMD.

Most experts expressed no concerns about recommending aflibercept 8 mg in newly diagnosed patients, but a minority cited clinical scenarios where caution may be warranted based on their clinical experiences. These included cases of advanced glaucoma, large pigment epithelial detachments, recent cardiovascular events or a history of intraocular inflammation. Aflibercept 8 mg has demonstrated a safety profile similar to that of aflibercept 2 mg in the PULSAR and PHOTON trials, and pooled data from the PULSAR, PHOTON and CANDELA trials showed no increase in IOI-related events compared with aflibercept 2 mg in the outcomes assessed [25, 26, 46]. Furthermore, while higher injection volumes may influence acute IOP dynamics, longer-acting anti-VEGF therapies that enable fewer injections over time may reduce cumulative stress on the optic nerve head. As such, no clinically significant differences in IOP have been observed between aflibercept 2 mg and 8 mg [20, 25, 47, 48] – experts noted that patients with advanced glaucoma may benefit from consideration of specialist glaucoma management prior to initiation of intravitreal injections. In some cases, digital eye globe massage, slow injection speed, prophylactic IOP-lowering medication or anterior chamber paracentesis may be considered [49, 50]. Experts acknowledged that, despite the reassuring safety profile observed in pivotal clinical trials, continued vigilance for sterile intraocular inflammation remains important, with emerging reports suggesting that such events can be uncommon but warrant prompt recognition and appropriate management [31, 51, 52]. Furthermore, as clinical experience with aflibercept 8 mg in routine clinical practice grows, evidence from real-world studies, such as the ongoing phase 4 SPECTRUM study, may help to mitigate such concerns as broader, real-world experience accumulates [27–31].

Experts agreed that aflibercept 8 mg is a reasonable switching option for previously treated patients, with timing guided by individual patient profiles and their own clinical judgement. The rationale for switching clustered around three clinical goals considered integral by the experts: enhancing durability; improving efficacy; and stabilising disease in patients with suboptimal responses. Aflibercept 8 mg has a demonstrated capacity to extend dosing intervals in previously treated patients [53]. Experts suggested that in patients with well-controlled disease, aflibercept 8 mg provides an opportunity to maintain stability while meaningfully reducing treatment burden. For patients who are stable on shorter intervals (e.g. every 8 weeks), switching can help secure long-term control by gradually extending intervals, while most experts noted that patients already on 8–12-week intervals may still benefit from extending to 16–24 weeks. Based on the available published literature and their own clinical observations in practice, the experts strongly supported the use of aflibercept 8 mg to improve anatomical response and achieve stability in poorly controlled patients who require frequent injections or have persistent fluid despite treatment, thereby enabling interval extension once disease control is achieved. In both groups, decisions can be individualised and guided with appropriate monitoring.

Some divergence of opinion was observed regarding treatment-initiation and interval-modification strategies, reflecting the diversity of real-world practice where decisions are tailore d to individual patient response and may be influenced by clinic capacity and healthcare system constraints. Experts also noted that the durability profile of aflibercept 8 mg, together with learnings from recent trial designs, may prompt modification of established routine care protocols, such as the confidence to implement earlier and larger interval extensions. While most experts supported extending intervals in 4-week increments, a minority favoured 2-week increments in complex or unstable cases based on their clinical experiences. Similarly, while some advocated monitoring only at the time of injection, others preferred interim visits for high-risk or newly extended patients.

As with all expert-driven work, the recommendations should be interpreted alongside the growing body of real-world evidence and are based on the currently available data and clinical experience, which may evolve as additional evidence emerges over time. While early findings from the ongoing, real-world SPECTRUM study have already shown visual acuity gains in treatment-naïve patients with nAMD, together with improvements in central retinal thickness and retinal fluid outcomes in both treatment-naïve and previously treated cohorts, further reports of the real-world experience with aflibercept 8 mg are expected [27, 31]. In addition, other recently emerging real-world data in treatment-naïve and pre-treated eyes have shown anatomical improvements, durability and a favourable safety profile [54–62]. Collectively, these findings may suggest that the rapidly expanding real-world evidence base supports the broad clinical utility of aflibercept 8 mg across diverse clinical settings, although certainly longer term data is required to further substantiate this understanding.

This article draws on the expertise of 16 internationally recognised key opinion leaders from 12 countries, offering deep clinical experience. A structured iterative consultation process was employed; however, it should be noted that a formal Delphi methodology was beyond the scope of this work and participating experts were not selected through a predefined or systematic process. The expert opinions presented reflect the interpretation of selected evidence and clinical experience of the authors and do not represent the positions of any professional society or organisation and may not be representative of all retina specialists involved in the management of nAMD and DMO globally.

In conclusion, this paper reports expert interpretation of clinical and growing real-world evidence for the use of aflibercept 8 mg for nAMD and DMO from a group of 16 retinal specialists. The recommendations presented aim to provide expert insights that improve physician confidence in delivering personalised treatment pathways for patients in clinical settings. Ongoing real-world studies and longer-term follow-up will further define the optimal positioning of aflibercept 8 mg within increasingly individualised treatment paradigms.

## Supplementary information

Supplementary Table 1. 8mg Expert Opinion_Supplementary information
