# Correction: Aflibercept 8 mg in neovascular age-related macular degeneration and diabetic macular oedema: Translating clinical trials into clinical practice

**DOI:** 10.1038/s41433-026-04887-2

**Published:** 2026-10-20

**Authors:** Sobha Sivaprasad, Michael W. Stewart, Charles C. Wykoff, David Eichenbaum, Jean-Francois Korobelnik, Paolo Lanzetta, Varun Chaudhary, Chui Ming Gemmy Cheung, Richard Gale, Marion R. Munk, Taiji Sakamoto, Andreas Stahl, Javier Zarranz-Ventura, Tien Yin Wong, Frank G. Holz, Anat Loewenstein

**Affiliations:** 1 NIHR Biomedical Research Centre, Moorfields Eye Hospital NHS Foundation Trust, London, UK; 2 Department of Ophthalmology, Mayo Clinic, Jacksonville, FL USA; 3 Retina Consultants of Texas, Retina Consultants of America, Houston, TX USA; 4 Retina Vitreous Associates of Florida, Saint Petersburg, FL USA; 5 Morsani College of Medicine at the University of South Florida, Tampa, FL USA; 6 CHU Bordeaux, Service d’Ophtalmologie, Bordeaux, France; 7 University of Bordeaux, INSERM, BPH, UMR1219 Bordeaux, France; 8 Department of Medicine-Ophthalmology, University of Udine, Udine, Italy; 9 Istituto Europeo di Microchirurgia Oculare-IEMO, Udine, Italy; 10 Department of Health Research Methods, Evidence and Impact, McMaster University, Hamilton, ON Canada; 11 Division of Ophthalmology, Department of Surgery, McMaster University, Hamilton, ON Canada; 12 Singapore Eye Research Institute, Singapore National Eye Centre, Singapore, Singapore; 13 Duke-National University of Singapore Medical School, Singapore, Singapore; 14 Hull York Medical School, University of York, York, UK; 15 Hull York Medical School, University of York, and York and Scarborough Teaching Hospitals NHS Foundation Trust, York, UK; 16 Gutblick Practise Group, Pfäffikon, Switzerland; 17 Department of Ophthalmology, Inselspital, Bern University Hospital, University of Bern, Bern, Switzerland; 18 Department of Ophthalmology, Northwestern University, Feinberg School of Medicine, Chicago, IL USA; 19 Department of Ophthalmology, Kagoshima University, Kagoshima, Japan; 20 Department of Ophthalmology, University Medicine Greifswald, Mecklenburg-Vorpommern, Greifswald, Germany; 21 Hospital Clínic de Barcelona, Barcelona, Spain; 22 Department of Surgery and Medical-Surgical Specialties, Faculty of Medicine and Health Sciences, Universitat de Barcelona, Barcelona, Spain; 23 Institut d’Investigacions Biomèdiques August Pi i Sunyer (IDIBAPS), Barcelona, Spain; 24 Beijing Visual Science and Translational Eye Research Institute (BERI), Beijing Tsinghua Changgung Hospital, Beijing, School of Clinical Medicine, Tsinghua Medicine, Tsinghua University, Beijing, China; 25 School of Biomedical Engineering, Tsinghua Medicine, Tsinghua University, Beijing, China; 26 Department of Ophthalmology and GRADE Reading Center, University of Bonn, Bonn, Germany; 27 Department of Ophthalmology, Tel Aviv Medical Centre, Tel Aviv, Israel; 28 Gray Faculty of Medical and Health Sciences, Tel Aviv University, Tel Aviv, Israel

**Keywords:** Therapeutics, Drug therapy

Correction to: *Eye* https://doi.org/10.1038/s41433-026-04779-5, published online 07 August 2026

In this article typos in text, references, funding and competing interest section have been corrected.

**Figure:**
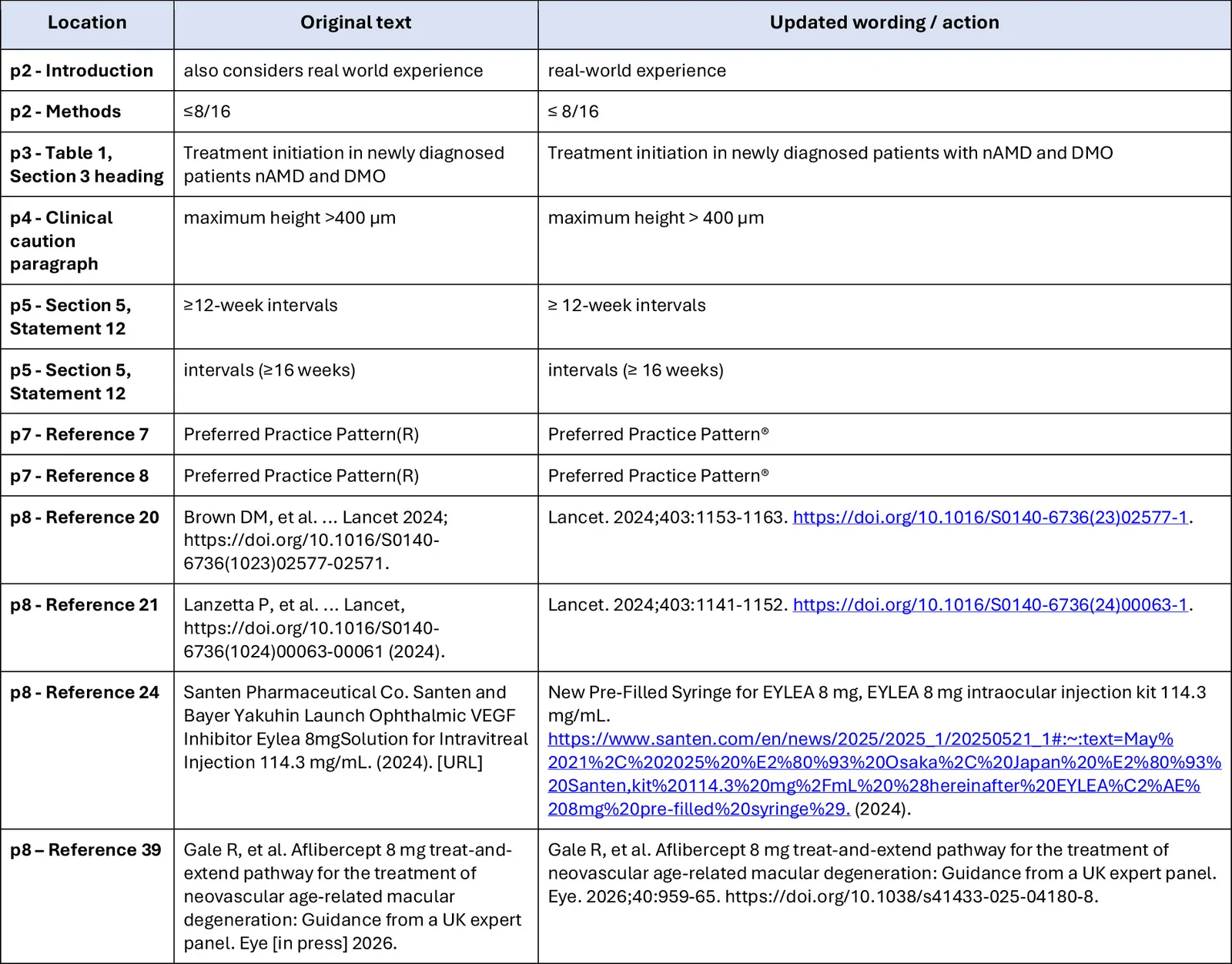

**Figure:**
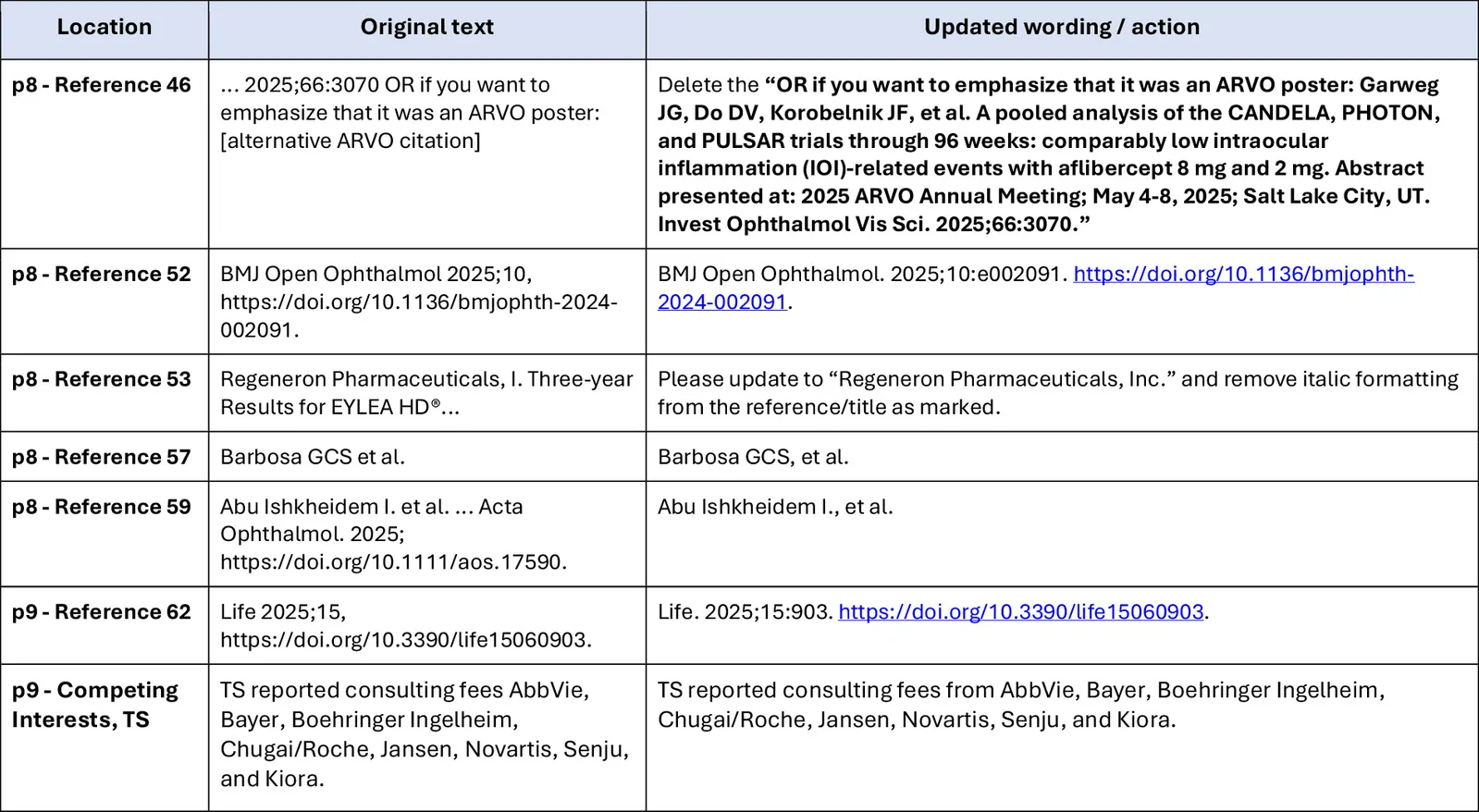

The original article has been corrected.

