# Supplementary material for: Aflibercept 8 mg in neovascular age-related macular degeneration and diabetic macular oedema: Translating clinical trials into clinical practice
Source: Eye (Lond). 2026 Aug 7;40(16):2507–15. doi: 10.1038/s41433-026-04779-5 (PMC13754902; doi:10.1038/s41433-026-04779-5)
Supplement: Supplementary file 1 [file 41433_2026_4779_MOESM1_ESM.docx]

**Supplementary Information**

**Text summary** (words: 17 [max 50]): The Within3 survey questions posed to experts and corresponding quantitative results are presented in Supplementary Table 1.

**Supplementary Table 1. Within3 survey questions and quantitative results**

| **SECTION 1: Newly diagnosed patients with nAMD and DME** | |
| --- | --- |
| Do you agree with the recommendation “Aflibercept 8 mg can be considered as a potential first-line treatment in all treatment-naïve patients with nAMD and DME”? | 100% agreed |
| Please explain if there are any newly diagnosed patients in whom you would be cautious to initiate treatment with aflibercept 8 mg. Please provide the rationale for your answer, based on your clinical experience and the available data | Open-text response |
| **SECTION 2: Previously treated patients with nAMD and DME** | |
| Do you agree with the recommendation “Aflibercept 8 mg can be considered an option to switch to for all patients with nAMD or DME, and therefore all patients may be informed about the possibility of switching to aflibercept 8 mg” | 100% agreed |
| Please explain what factors you would consider when deciding on the timing of the switch to aflibercept 8 mg, based on your clinical experience and the available data | Open-text response |
| Please indicate if you agree with the rationale for switching to aflibercept 8 mg for the patient profile below. |  |
| Well-controlled disease (stable vision, no fluid), with dosing intervals less than every 8 weeks. A switch to aflibercept 8mg in this profile could extend their treatment intervals whilst maintaining disease control, thus reducing treatment burden | 100% agreed |
| Well-controlled disease (stable vision, no fluid), with dosing intervals of 8–12 weeks or longer. A switch to aflibercept 8 mg in this profile could *further* extend their treatment intervals, whilst maintaining disease control, thus *further* reducing treatment burden | 94% agreed |
| Poorly controlled disease (fluctuating vision and/or fluid), with dosing intervals less than every 8 weeks. A switch to aflibercept 8 mg in this profile could optimise disease control and also potentially increase treatment intervals, thus improving patient outcomes | 94% agreed |
| Please explain if there are any patient profiles that are currently missing from [this] list, particularly if they are relevant when discussing a switch to aflibercept 8 mg | Open-text response |
| **SECTION 3: Treatment initiation in newly diagnosed patients with nAMD and DME** | |
| Do you recommend that all newly diagnosed patients should receive three initial monthly doses of aflibercept 8 mg upon its initiation? | 56% voted yes |
| At what stage during treatment initiation with aflibercept 8 mg do you decide on when to modify the treatment interval of your patient?  4 weeks after the *second* monthly injection  4 weeks after the *third* monthly injection  At another time (please specify below) | Voting was split  31%  31%  38% |
| **SECTION 4: Initiation of aflibercept 8 mg in previously treated patients with nAMD and DME** | |
| Do you agree with the recommendation “When switching patients to aflibercept 8 mg, initiation on the same treatment interval as their previous agent may be considered”? | 94% agreed |
| Do you agree that only a single parameter should be changed at a time when considering switching to aflibercept 8 mg? *For example, if the agent is switched, the treatment interval should be kept the same* | 94% agreed |
| In patients with well controlled disease (stable vision, no fluid) who are being switched from aflibercept 2 mg to aflibercept 8 mg, do you agree that the patient’s treatment interval can be extended immediately after the first aflibercept 8 mg injection and that no initial monthly doses are needed? | 81% voted yes |
| In patients with poorly controlled disease (fluctuating vision and/or fluid) who are switched to aflibercept 8 mg due to a lack of efficacy with their prior treatment, would you consider either:  Three initial monthly doses of aflibercept 8 mg  Decisions on the dosing of aflibercept 8 mg to be made each month  Another treatment approach (please specify) | Voting was split:  38%  50%  13% |
| **SECTION 5: Treatment response and dose regimen modification** | |
| Do you agree with the recommendation “Treatment intervals can be extended in 4-week increments, with the option to reduce intervals in 2-week increments; additional monitoring visits may be considered with treatment intervals ≥16 weeks”? | 81% agreed |
| Please explain if there are any patients in whom you would feel confident to extend immediately to 16‑week intervals after three initial monthly doses with aflibercept 8 mg. Please provide the rationale for your answer, based on your clinical experience and the available data | Open text response |
| Do you agree that OCT serves as the key measure on which treatment decisions are based, such as interval adjustment? | 100% agreed |
| Please describe how often you would monitor a patient if their treatment intervals were extended to 20-weekly or 24-weekly? Please provide the rationale for your answer, based on your clinical experience and the available data | Open text response |
| **SECTION 6: Patient–physician dialogue** | |
| Do you agree with the recommendation “When discussing with treatment-naïve patients the option to begin with aflibercept 8 mg, it is important to clearly explain the rationale behind the proposal”? | 100% agreed |
| Please suggest any materials and pertinent points that will help you discuss the rationale for starting with aflibercept 8 mg with your treatment-naïve patient | Open text response |
| Do you agree with the recommendation “When discussing with patients the option to switch to aflibercept 8 mg, it is important to clearly explain the rationale behind the proposal”? | 100% agreed |
| Please suggest materials that will help you discuss with your patient the switch to aflibercept 8 mg from other agents, including aflibercept 2 mg | Open text response |

Note, percentages may not add up to 100% due to rounding. DME, diabetic macular oedema; nAMD, neovascular age-related macular degeneration.
